# An engineered protein antagonist of K-Ras/B-Raf interaction

**DOI:** 10.1038/s41598-017-05889-7

**Published:** 2017-07-19

**Authors:** Monique J. Kauke, Michael W. Traxlmayr, Jillian A. Parker, Jonathan D. Kiefer, Ryan Knihtila, John McGee, Greg Verdine, Carla Mattos, K. Dane Wittrup

**Affiliations:** 1 Department of Chemical Engineering, Massachusetts Institute of Technology, Cambridge, MA 02139 USA; 2 Koch Institute for Integrative Cancer Research, Massachusetts Institute of Technology, Cambridge, MA 02139 USA; 3 Department of Chemistry and Chemical Biology, Northeastern University, Boston, Massachusetts 02115 USA; 4 Department of Chemistry and Applied Biosciences, Institute of Pharmaceutical Sciences, Swiss Federal Institute of Technology, Zurich, Switzerland; 5 Department of Chemistry and Chemical Biology, Harvard University, Cambridge, MA 02138 USA; 6 Department of Stem Cell and Regenerative Biology, Harvard University, Cambridge, MA 02138 USA; 7 Department of Molecular and Cellular Biology, Harvard University, Cambridge, MA 02138 USA; 8 Department of Biological Engineering, Massachusetts Institute of Technology, Cambridge, MA 02139 USA

## Abstract

Ras is at the hub of signal transduction pathways controlling cell proliferation and survival. Its mutants, present in about 30% of human cancers, are major drivers of oncogenesis and render tumors unresponsive to standard therapies. Here we report the engineering of a protein scaffold for preferential binding to K-Ras G12D. This is the first reported inhibitor to achieve nanomolar affinity while exhibiting specificity for mutant over wild type (WT) K-Ras. Crystal structures of the protein R11.1.6 in complex with K-Ras WT and K-Ras G12D offer insight into the structural basis for specificity, highlighting differences in the switch I conformation as the major defining element in the higher affinity interaction. R11.1.6 directly blocks interaction with Raf and reduces signaling through the Raf/MEK/ERK pathway. Our results support greater consideration of the state of switch I and provide a novel tool to study Ras biology. Most importantly, this work makes an unprecedented contribution to Ras research in inhibitor development strategy by revealing details of a targetable binding surface. Unlike the polar interfaces found for Ras/effector interactions, the K-Ras/R11.1.6 complex reveals an extensive hydrophobic interface that can serve as a template to advance the development of high affinity, non-covalent inhibitors of K-Ras oncogenic mutants.

## Introduction

GTPases K-Ras, H-Ras, and N-Ras comprise the most frequently mutated family of oncoproteins in human cancers, including three of the most lethal forms, cancers of the lung, colon, and pancreas. Known to initiate cell transformation and drive oncogenesis, mutant Ras proteins have been shown to promote tumor maintenance as well. Given the high level of incidence across a large subset of cancer types and the well-established role of Ras in tumor initiation, development, and progression, a large effort in Ras inhibitor development has been put forth^1–3^.

Despite decades of research, however, no drugs directly targeting Ras are currently available. This is primarily due to its disordered active site and smooth surface lacking well-defined drug-binding pockets^2, 3^. Mutations impair intrinsic Ras activity^4^, preventing GTP hydrolysis and resulting in constitutively active Ras capable of binding effector proteins including Raf^5^ and PI3K^6^. Mutational activation of Ras proteins and the subsequent constitutive signaling downstream drives uninhibited proliferation and promotes migration and invasion. The challenge of targeting Ras pharmacologically is compounded by difficulty in attaining drug specificity for mutant over wild type protein and the fact that each mutant is likely to function by unique mechanisms^2^. Here we present an inhibitor R11.1.6 engineered on a scaffold based on the thermostable protein Sso7d for preferential binding to K-Ras G12D and reveal an extensive hydrophobic interface on K-Ras that can be exploited in future inhibitor development.

## Results

### Engineering and characterization of mutant K-Ras specific protein binder R11.1.6

**Figure 1:**
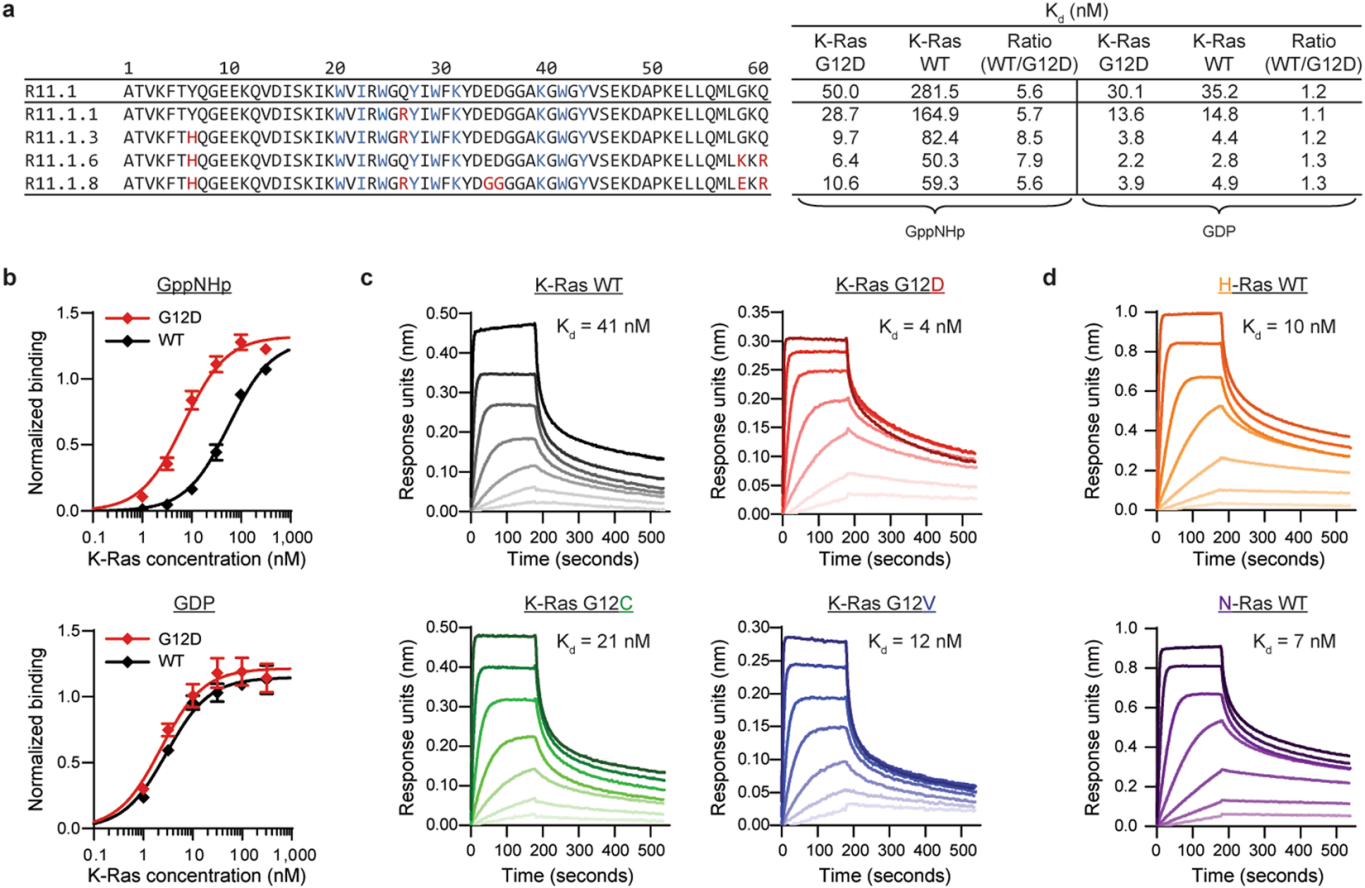
Engineered Sso7d protein selectively binds mutant K-Ras. (**a)** Amino acid sequences of parental binder R11.1 and affinity-matured clones. The nine residues of the Sso7d binding surface are depicted in blue; R11.1 framework mutations are shown in red. Dissociation constants (K_d_) obtained from yeast surface display (YSD) titrations detected using flow cytometry are given on the right. (**b)** YSD titrations of R11.1.6 with K-Ras loaded with GDP or the non-hydrolyzable GTP analog GppNHp. Error bars represent SEM of n = 3 independent binding experiments. (**c**,**d)** Binding of R11.1.6 to immobilized GppNHp-loaded K-Ras, H-Ras, or N-Ras measured using bio-layer interferometry. Concentrations of R11.1.6 curves from dark to light: 1000, 333.3, 111.1, 37, 12.3, 4.1, 1.4 nM. K_d_ values were calculated from steady-state values.

The recent success of allele-specific inhibitors for K-Ras G12C^7, 8^ prompted us to target the G12D mutation, present in approximately 50% of K-Ras-driven pancreatic and colorectal cancers^3^. We recently showed that charge-neutralized variants of the Sso7d protein from the hyperthermophilic archaeon *Sulfolobus solfataricus* can be engineered to bind targets with high affinity and specificity^9^. Because of its small size (7 kDa), high thermostability (T_m_ of 98 °C), and lack of cysteines and glycosylation sites, the Sso7d scaffold is well suited for targeting an intracellular protein with a cytoplasmically expressed antagonist. Using yeast surface display^10^, we isolated R11.1 to preferentially bind GppNHp-loaded K-Ras G12D over WT (see Methods). Affinity maturation of R11.1 yielded four unique clones with varying degrees of affinity and specificity (Fig. 1a). We chose to further pursue R11.1.6, which binds K-Ras G12D in the GppNHp-bound state with single-digit nanomolar affinity – eight-fold greater than for the wild type. To our knowledge, this is the first inhibitor with such high affinity for mutant K-Ras as well as specificity over the wild type protein.

Intriguingly, the mutant vs. wild type specificity, but not high affinity, is lost in the GDP-bound state (Fig. 1b). This was observed for the parental R11.1 and the remaining affinity-matured clones as well (Extended Data Fig. 1). The loss of mutation-dependent binding suggests specificity is due to the conformation of GppNHp-bound K-Ras G12D, rather than the mutation itself. We therefore evaluated binding to K-Ras mutants G12C and G12V using bio-layer interferometry and found that R11.1.6 binds both mutants with an affinity comparable to K-Ras G12D (Fig. 1c). Given the high degree of homology between Ras isoforms K-Ras, H-Ras, and N-Ras, which share 100% sequence identity in the effector lobe (residues 1–86) and greater than 90% identity in the allosteric lobe (residues 87–166)^11^, we expected binding of R11.1.6 to H- and N-Ras as well. Indeed, low nanomolar affinity was measured for both (Fig. 1d).

### Co-crystal structures of R11.1.6 and K-Ras G12D and WT

**Figure 2:**
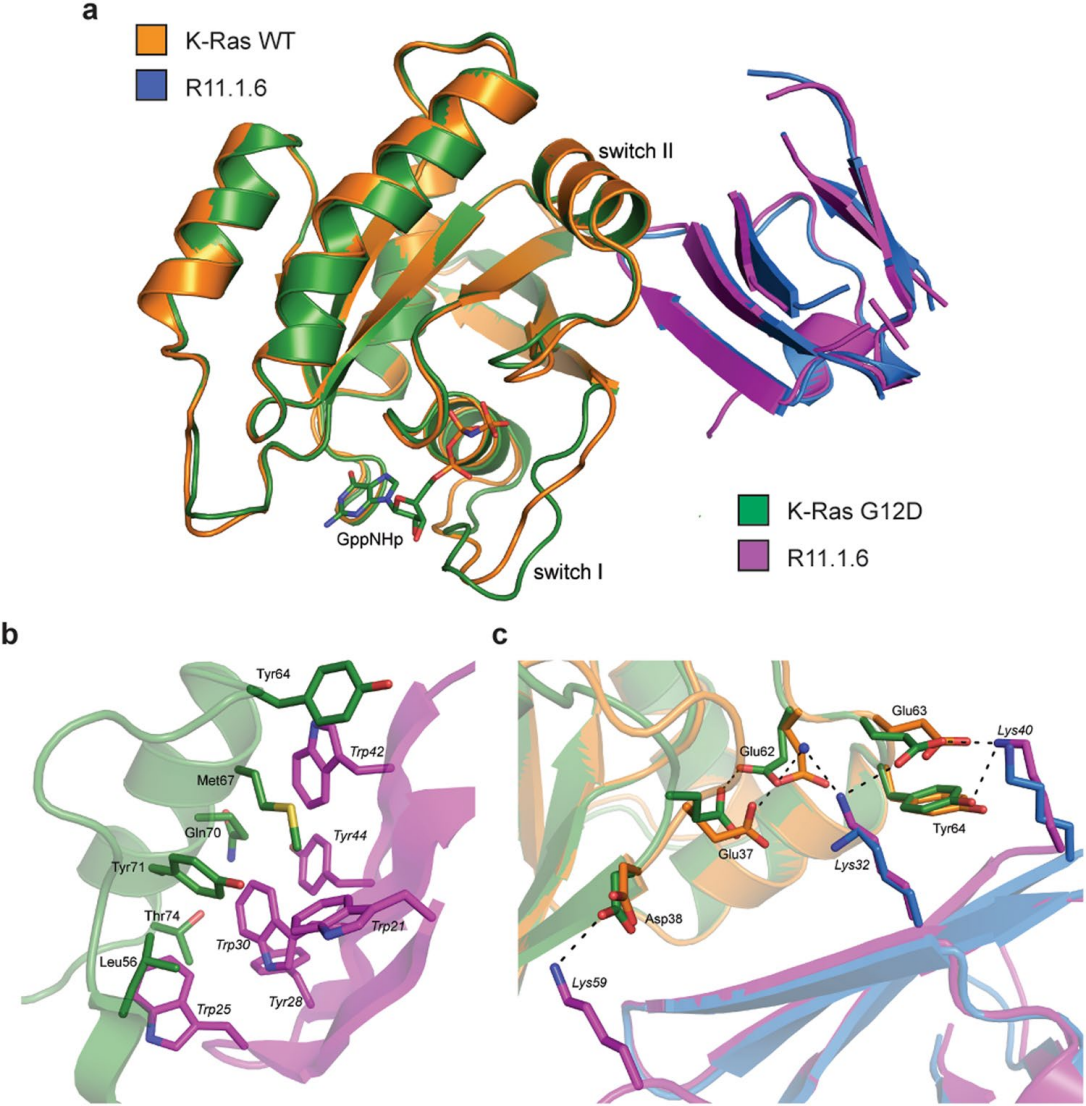
Switch I conformation gives rise to R11.1.6 mutant-specific binding. (**a**) Overlay of co-crystal structures of R11.1.6 with GppNHp-bound K-Ras WT and G12D. The C-terminus of R11.1.6 in both structures is partially disordered. (**b**) Binding interface between R11.1.6 (magenta) and K-Ras G12D (green) at switch II, highlighting the hydrophobic pocket created upon complex formation. K-Ras G12D residues are indicated in regular font; R11.1.6 residues are shown in italics. (**c**) Interactions between R11.1.6 lysine residues and K-Ras that give rise to greater affinity for the G12D mutant over WT. Bond distances of less than 4.0 Å are shown in dashed lines. A water molecule is shown as a blue sphere.

To better understand their molecular interactions, we obtained co-crystal structures of R11.1.6 bound to GppNHp-loaded K-Ras G12D and to GppNHp-loaded K-Ras WT at resolutions of 2.20 and 2.30 Å, respectively (Fig. 2a and Extended Data Table 1). Only a few crystal structures of K-Ras in its active conformation have been obtained^12, 13^, and none in complex with another protein, making this a useful step in studying active K-Ras interaction with other proteins and in potentially developing future inhibitors via a structure-guided approach. The asymmetric unit of the K-Ras G12D/R11.1.6 complex contains four molecules, with distinct R11.1.6 molecules interacting at the switch I or switch II regions of K-Ras (Extended Data Fig. 2a). Binding at switch I appears to be an artifact of crystal contacts consisting of a few polar interactions and does not significantly contribute to the measured nanomolar affinity (Extended Data Fig. 2b). For both mutant G12D and WT structures, the K-Ras/R11.1.6 interface consists of switch II in a conformation that selectively exposes its hydrophobic residues, which are intercalated by a series of aromatic R11.1.6 residues. The surfaces of the two proteins are highly complementary, completely excluding solvent molecules (Fig. 2b). Interestingly, the R11.1.6 Trp25 indole side chain directly overlays with the indole moieties of small molecules previously identified to bind to K-Ras^14^ (Extended Data Fig. 3). This unique convergence of a small molecule screen and directed protein evolution to the same K-Ras binding site demonstrates the potential of this interface for future inhibitor development.

The structures show R11.1.6 makes contact with K-Ras away from the GTP-binding active site, confirming that the mutant specificity is indeed due to the mutant’s conformation rather than direct contact with the mutated residue. Switch I of GTP-bound Ras can be found in two major interconverting conformations, state 1 and state 2^15^. Recent NMR data showed that switch I in K-Ras G12D is stabilized in state 2, while WT K-Ras has a more open and dynamic active site, with significant population of state 1 (unpublished data). Our structural data mirror these results. In the G12D structure, switch I adopts a conformation resembling state 2, which places Glu37 and Glu63 in an orientation that favors interaction with R11.1.6 residues Lys32 and Lys40 at the periphery of the interface, providing additional stabilizing interactions to the complex (Fig. 2c). In contrast, this R11.1.6 “lysine claw” does not appear to be as strong in the WT structure, in which switch I exhibits a state 1 conformation and Lys32 is oriented away from Glu63. These differences in interaction between Lys40 and Lys32 and switch I residues appear to give rise to the greater specificity of R11.1.6 for the G12D mutant. Consistently, H-Ras, which is also stabilized in state 2^15^, has an affinity for R11.1.6 more similar to that obtained for K-Ras G12D than for WT K-Ras (Fig. 1). Presumably this change in conformation, and therefore the mutant specificity, is lost in the GDP-bound state.

### R11.1.6 inhibits intrinsic K-Ras activity and interaction with B-Raf

**Figure 3:**
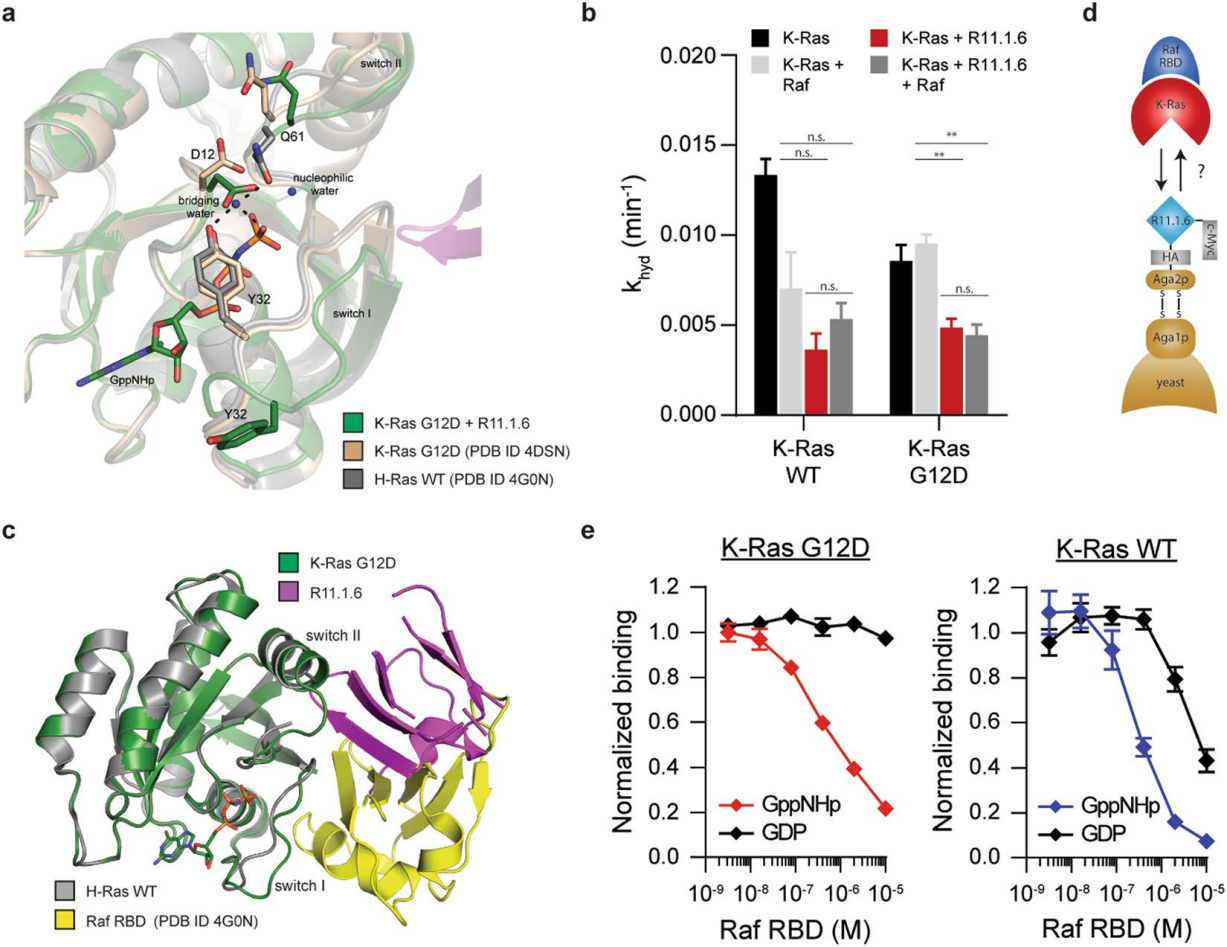
R11.1.6 reduces K-Ras intrinsic hydrolysis but directly competes with Raf. (**a**) Overlay of the co-crystal structure of R11.1.6 with K-Ras G12D with structures of K-Ras G12D in complex with a small molecule (PDB ID 4DSN) and H-Ras WT (PDB ID 4G0N), showing the disruption of Y32 and Q61 by R11.1.6, with an open switch I conformation. (**b**) Intrinsic hydrolysis rate constants (k_hyd_) of K-Ras alone or in the presence of R11.1.6 and/or the Ras binding domains (RBD and CRD, residues 51–196) of Raf. Error bars represent SEM of n = 3 independent experiments. **P < 0.01, 1-way ANOVA with Tukey post-test for analyzing all possible comparisons. (**c**) Overlay of the co-crystal structure of R11.1.6 with K-Ras G12D with the co-crystal structure of H-Ras WT with Raf RBD (PDB ID 4G0N). (**d**) Schematic representation of yeast surface display competition assay between R11.1.6 and Raf RBD for K-Ras binding. (**e**) Competition for binding of K-Ras as depicted in (**d**). Error bars represent SEM of n = 3 independent binding experiments.

Hydrolysis of GTP to GDP is required for termination of Ras signaling and is strongly dependent on GTPase-Activating Proteins (GAPs), which increase intrinsic hydrolysis rates by up to five orders of magnitude^16^. Because activating mutations such as those at codon 12 inhibit GAP-mediated hydrolysis, intrinsic GTPase activity becomes the determinant of the extent of output signaling in Ras-driven cancers^2^. The interaction between Ras active site residues Tyr32 and Gln61 and a bridging water molecule is critical for intrinsic GTP hydrolysis^17^. Binding of R11.1.6 at switch II alters the position of these residues, preventing the active site from reaching a catalytically competent state (Fig. 3a). For both K-Ras G12D and WT, the intrinsic hydrolysis rate is indeed significantly impaired by the presence of R11.1.6 (Fig. 3b).

Despite pharmacologically undesirable stabilization of K-Ras in its active state, the inhibitory potential of R11.1.6 is redeemed by its ability to directly block Ras interaction with downstream effector Raf (Fig. 3c). The pool of GTP-bound Ras can potentially be sequestered by R11.1.6 and therefore unable to signal. GTP hydrolysis rate constants for both K-Ras G12D and WT in the presence of R11.1.6 and a construct of Raf containing the two Ras binding domains (RBD and CRD) are consistent with the binder successfully competing with the effector protein, particularly in the case of the mutant (Fig. 3b). Crystallographic analysis further confirms the direct competition between Raf and R11.1.6 for Ras binding (Fig. 3c). To show this directly, we took advantage of yeast display technology, in which we measured binding of R11.1.6, expressed on the surface of yeast, to K-Ras pre-incubated with increasing concentrations of the Raf RBD (Fig. 3d). For both mutant G12D and WT K-Ras, the Raf RBD competed with R11.1.6 binding (Fig. 3e). As expected, nucleotide loading affected the extent of competition, since only GTP-bound Ras is able to bind Raf.

**Figure 4:**
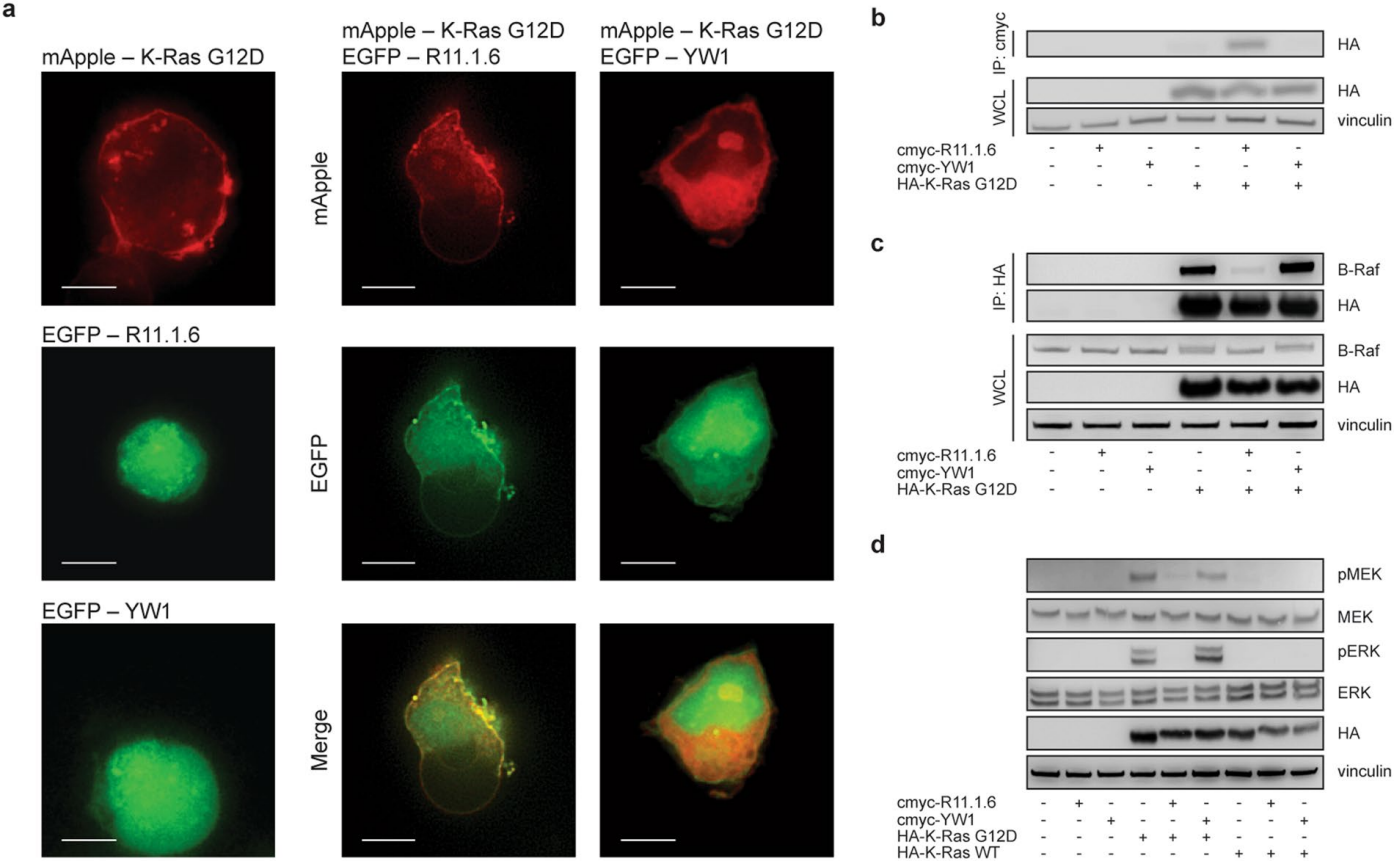
R11.1.6 specifically binds K-Ras G12D in cells, blocks K-Ras-B-Raf interaction, and inhibits signaling. (**a**) Co-localization of EGFP-R11.1.6 (green) with mApple-K-Ras G12D (red) in co-transfected HEK 293T cells. Scale bars are 10 μm. Images are representative of n = 2 biological replicates. Co-localization is quantified in Extended Data Figure 5a. (**b**) Co-immunoprecipitation of HA-tagged K-Ras G12D with cmyc-R11.1.6/YW1 in co-transfected HEK 293T cells, showing R11.1.6 specificity for K-Ras G12D. IP, immunoprecipitation; WCL, whole cell lysate. Results are representative of n = 2 biological replicates. (**c**) Co-immunoprecipitation of endogenous B-Raf with HA-tagged K-Ras G12D in co-transfected HEK 293T cells, showing inhibition of K-Ras-B-Raf binding by R11.1.6. Results are representative of n = 3 biological replicates. (**d**) Effect of R11.1.6 on phosphorylation of endogenous MEK (pMEK) and ERK (pERK) via HA-K-Ras G12D-induced signaling in co-transfected HEK 293T cells, showing inhibition of signaling by R11.1.6. Results are representative of n = 3 biological replicates. Quantification is provided in Extended Data Figure 5e. Full-length blots of the cropped ones shown here are given in Extended Data Figures 6, 7, and 8.

While clinical translation of R11.1.6 requires either an effective cytoplasmic delivery route or targeted gene therapy (both significant pharmacological challenges), it is nevertheless immediately useful as a genetically encoded tool for studying the consequences of Ras antagonism. We examined Raf inhibition and downregulation of cell signaling by R11.1.6 expression. Enhanced green fluorescent protein (EGFP) fused to R11.1.6 or a scrambled control protein YW1 (Extended Data Fig. 4a,b), in which Tyr28 and Trp30 of the parental R11.1 are swapped to eliminate binding to K-Ras (Extended Data Fig. 4c), was co-transfected with mApple-tagged K-Ras G12D into HEK 293T cells. Co-localization on the plasma membrane was only observed between R11.1.6 and K-Ras G12D and not the YW1 control (Fig. 4a and Extended Data Fig. 5a). Specific binding in cells is further shown by R11.1.6’s ability to pull down mutant K-Ras G12D (Fig. 4b). Non-specific interactions between R11.1.6 and other intracellular proteins are minimal, though we show the binding of R11.1.6 to WT Ras to be comparable to mutant (Extended Data Fig. 5b).

Co-immunoprecipitation of mutant K-Ras and B-Raf is inhibited only by R11.1.6 and not the scrambled control (Fig. 4c), extending our yeast display competition findings (Fig. 3e) to the mammalian cytosol. This direct competition with Raf translates to inhibition of Ras-Raf signaling through the mitogen-activated protein (MAP) kinase cascade (Fig. 4d). The extent of signal downregulation is directly related to the concentration of R11.1.6 in the cells (Extended Data Fig. 5c). Intriguingly, we did not observe a change in signaling through the PI3K/AKT/mTOR pathway (Extended Data Fig. 5d). The effect of Raf inhibition on PI3K/AKT signaling has been shown to depend on the mutational status of Ras and Raf. Cells with mutant Ras observed either no change or an increase in phosphorylated AKT (pAKT) in response to Raf inhibition, while those harboring the wild type protein responded with a decrease in pAKT^18^. It is possible that R11.1.6 blockade of Ras-Raf interaction parallels direct Raf inhibition and results in a similar feedback response through the PI3K/AKT pathway. Because compensatory mechanisms and cross-talk between pathways are strongly dependent on cell lines and Ras mutation status^19^, and since overexpression of mutant K-Ras does not incrementally increase pAKT levels in HEK 293T cells (Extended Data Fig. 5d), it is also likely that the lack of pAKT downregulation by R11.1.6-mediated Ras inhibition is related to the model system used.

## Discussion

Using directed evolution of a novel protein scaffold, we have engineered R11.1.6 to specifically and tightly bind oncogenic K-Ras G12D. Through crystallographic analysis of complexes between R11.1.6 and both mutant G12D and WT K-Ras we have identified the source of specificity and gained insight into the importance of the state of switch I. Given its dynamic nature, the conformation of switch I in WT K-Ras is likely in a constant state of flux, but mutant K-Ras G12D appears to strongly favor state 2 (unpublished data). This could be exploited in the design of inhibitors to obtain improved mutant specificity and also reveal new pockets in the otherwise smooth protein, as we have shown here.

Clinical Ras inhibition continues to be met with challenges. While a number of small molecules have been developed to directly block interaction of Ras with downstream effector Raf^20–22^, the micromolar affinity and small size of these compounds renders them not very potent. Peptide and protein-based Ras inhibitors have also been explored. Cyclic peptides^23^ as well as stapled SOS1 peptides^24^ have been shown to bind with double-digit nanomolar affinity, a single antibody domain was shown to exhibit preferential mutant binding^25^, and a monobody was isolated with nanomolar affinity to H- and K-Ras, but not N-Ras^26^. Recently, adoptive cell transfer of K-Ras G12D specific CD8+ T cells resulted in regression of metastatic lesions in a patient^27^, shedding light on the power of the immune system in targeting Ras-driven cancers. Despite this success, the Ras problem is far from solved.

R11.1.6 is unique in its extensive hydrophobic interface with K-Ras. None of the above protein molecules interacts primarily with switch II and those for which there is structural information reveal a polar interface either with switch I^23–25^ or elsewhere^26^, particularly difficult to mimic in drug development. Small molecules do interact at switch II making primarily hydrophobic contacts, but the interface is small and the binding weak^12, 14^. R11.1.6 explores that surface extensively. Thus, R11.1.6 can serve as a novel tool to study Ras biology, and the structure of the complex provides important molecular clues for how to modulate switch II to interfere with the Ras/Raf interaction in the development of new inhibitors.

## Methods

### Reagents

Detection reagents for yeast display were mouse-anti-c-MYC (clone 9E10, 13–2500), goat anti-chicken-AF 488 (A-11039), goat anti-mouse-AF 488 (A-11001), and Streptavidin-AF 647 (S-32357) from Life Technologies; chicken-anti-c-MYC (ACMYC) from Gallus Immunotech Inc.; and anti-His-AF 647 (35370) from Qiagen. Western blot antibodies were anti-vinculin (13901S), anti-B-Raf (14814S), anti-pMEK1/2 (Ser217/221) (9121S), anti-MEK1/2 (9122S), anti-pERK p44/42 (Thr202/Tyr204) (9101S), anti-ERK (9102S), anti-pAKT (Ser473) (9271S), and anti-AKT (9272S) from Cell Signaling Technologies, and anti-HA (16B12) from BioLegend. Blots were detected with HRP-conjugated secondaries (406401 and 405306) from BioLegend. GppNHp and GDP were purchased from Abcam, Jena Bioscience, and Sigma-Aldrich.

### Cell culture

HEK 293T cells (ATCC) were cultured in DMEM with 10% FBS. DMEM was purchased from ATCC.

### Subcloning and transfections

Cmyc-R11.1.6, cmyc-YW1, HA-K-Ras G12D, HA-K-Ras WT, EGFP-R11.1.6, EGFP-YW1, and mApple-K-Ras G12D were cloned into the gWIZ vector (Genlantis) for mammalian expression using In-Fusion Cloning (Clontech) according to the manufacturer’s instructions. The sequence for R11.1.6 was PCR amplified from the yeast display vector (pCTCON2), digested with BsaI and XbaI, and cloned into BsaI digested pE-SUMO-vector (LifeSensors) for bacterial protein expression. To make the scrambled YW1 control and R11.1.6 binding interface alanine point mutants, the QuikChange site-directed mutagenesis method (Agilent) was used according to the manufacturer’s instructions. Transient transfections into HEK 293T cells were carried out using calcium phosphate. Briefly, DNA diluted in water was added to 2 M CaCl2, to which 2x HBS was added dropwise. The transfection mixture was added to plated cells and incubated for 8 hours, after which the transfection medium was replaced with complete medium. Unless indicated otherwise, the ratio of DNA transfected for K-Ras constructs to R11.1.6/YW1 constructs was 1:4.

### Protein expression and purification

R11.1.6 and YW1 were expressed as fusion proteins consisting of an N-terminal hexahistidine tag, followed by small ubiquitin-like modifier (SUMO) and Sso7d, using the pE-SUMO-vector (LifeSensors), as previously described^9^. The proteins were produced in Rosetta 2 (DE3) *E. coli* cells and purified using TALON Metal Affinity Resin (Clontech). For crystallization, SUMO-R11.1.6 was digested with the protease Ulp1 (SUMO protease 1), resulting in cleavage of the N-terminal His6-SUMO-tag right before the N-terminus of R11.1.6. After overnight digestion at 22 °C, the digestion product was purified using TALON Metal Affinity Resin. Digested SUMO, non-digested SUMO-fusion proteins, and SUMO protease 1 (all containing a hexahistidine tag) bound to the resin. The flow-through, only containing R11.1.6, was collected, buffer-exchanged to crystallization buffer (20 mM HEPES, 50 mM NaCl, 20 mM MgCl2, pH 7.5), and concentrated to ~22 mg/mL.

K-Ras WT, G12D, G12C, G12V, H-Ras, N-Ras (G domain, residues 1–166), the Raf RBD (residues 51–131), and the Raf construct of RBD and CRD (residues 51–196) were expressed in BL21(DE3) *E. coli* cells and purified as described previously^28, 29^. Nucleotide exchange was carried out using alkaline phosphatase beads (Sigma-Aldrich) to provide Ras proteins bound to a non-hydrolyzable GTP analog, guanosine 5′-[β,γ-imido]triphosphate (GppNHp). The purified proteins were concentrated to 20–30 mg/mL and stored at −80 °C in stabilization buffer (20 mM HEPES, 50 mM NaCl, 20 mM MgCl2, 1 mM DTT, 1% v/v glycerol, pH 7.5).

The K-Ras used for yeast display selections and titration experiments was expressed as a fusion protein consisting of an N-terminal hexahistidine tag, followed by small ubiquitin-like modifier (SUMO) and the first 166 amino acids of human K-Ras isoform 4B using the pE-SUMO vector. Both K-Ras WT and K-Ras G12D were expressed as His6-SUMO-fusions. The proteins were produced in Rosetta 2 (DE3) *E. coli* cells. After expression at 30 °C for 5 hours, cells were centrifuged and re-suspended in 40 mL HisBuffer 1 T (50 mM Tris/HCl, 300 mM NaCl, 10 mM imidazole, 5 mM MgCl2, pH 7.5) per 2 liters of culture volume, followed by addition of cOmplete EDTA-free protease inhibitor cocktail (Roche). Cells were lysed by sonication, followed by centrifugation (20,000 × g, 15 minutes, 4 °C). The supernatant was purified using TALON Metal Affinity Resin (Clontech). After three washing steps with HisBuffer 1 T, the protein was eluted with HisBuffer 2 T (50 mM Tris/HCl, 300 mM NaCl, 150 mM imidazole, 5 mM MgCl2, pH 7.5). Immediately following elution from the column, DL-Dithiothreitol (DTT; 1 mM final concentration) and cOmplete EDTA-free protease inhibitor cocktail solution (Roche) were added. The protein samples were purified on a Superdex 75 10/300 GL column (GE Healthcare), pre-equilibrated in Ras Phosphatase Buffer (32 mM Tris/HCl, 200 mM (NH4)2SO4, 1 mM DTT, 0.5 mM NaN3, 1 µM ZnCl2, pH 8.0). The purified protein was concentrated to 150–300 µM using Amicon Ultra Centrifugal Filter Units (EMD Millipore). After adding 0.5 µL calf intestinal alkaline phosphatase (New England Biolabs) per 100 µL of protein solution and GppNHp (Sigma-Aldrich) to three times the protein concentration, the K-Ras-solution was incubated at 22 °C for 90 minutes. Subsequently, the protein samples were purified on a Superdex 75 10/300 GL column pre-equilibrated in Ras Storage Buffer (20 mM HEPES, 100 mM NaCl, 5 mM MgCl2, 1 mM DTT, pH 7.5). After SEC purification, the samples were split into two parts, of which one was biotinylated using EZ-Link Sulfo-NHS-LC-Biotin (Life Technologies). Protein samples were supplemented with glycerol (10% final concentration), shock frozen in liquid nitrogen, and stored at −80 °C.

### Sso7d selections and characterization

K-Ras binders were selected from libraries rcSso7d-11 and rcSso7d-18^9^ using yeast display technology. All selections were conducted in Ras Selection Buffer (50 mM Tris/HCl, 100 mM NaCl, 5 mM MgCl2, 1 g/L BSA, 0.1 mM DTT, 100 nM GppNHp, pH 7.4) using His6-SUMO-K-Ras-G12D as antigen.

Initially, bead selections were conducted using streptavidin-coated Dynabeads (Thermo Fisher) and biotinylated K-Ras-G12D as described previously^10^. In total, two positive selections (binding to antigen-loaded beads) and three negative selections (incubation with unloaded beads and selection of non-bound cells) were done. The affinity of the bead selected libraries was improved by two rounds of affinity maturation, with each round consisting of error prone PCR and two rounds of magnetic bead selections. Error prone PCR was done by using the nucleotide analogs 8-oxo-2′-deoxyguanosine-5′-triphosphate (8-oxo-dGTP) and 2′-deoxy-p-nucleoside-5′-triphosphate (dPTP) (2 µM each; both from TriLink BioTechnologies) as described previously^10^. After the two rounds of affinity maturation, libraries were further selected by FACS. Briefly, washed cells were incubated with biotinylated SUMO-K-Ras-G12D and mouse anti-c-MYC (clone 9E10) followed by incubation with Streptavidin-AF 647 and goat anti-mouse IgG-AF 488. Cells were sorted on a FACS Aria IIU (BD Biosciences). After three rounds of FACS enrichment, individual clones were sequenced and analyzed for binding to K-Ras G12D and K-Ras WT, yielding the binder R11.1. Adding an excess of non-biotinylated SUMO did not block binding of biotinylated SUMO-K-Ras-G12D, confirming that R11.1 bound to K-Ras and not to SUMO.

In order to improve the affinity further, R11.1 was subjected to one more round of affinity maturation, consisting of error prone PCR and four rounds of FACS. Sequencing of single clones yielded R11.1.1, R11.1.3, R11.1.6, and R11.1.8.

To obtain binding dissociation constants, individual mutants were expressed on the surface of yeast and tested for binding to His6-SUMO-K-Ras G12D and His6-SUMO-K-Ras WT. Titrations were performed in Ras Selection Buffer in the presence of either 10 µM GppNHp or 10 µM GDP. Cells were incubated with various concentrations of non-biotinylated His6-SUMO-K-Ras and with chicken anti-c-MYC. After washing, cells were stained with goat anti-chicken IgY-AF 488 and with mouse anti-penta-His-AF 647 for detecting the His-tagged SUMO-K-Ras proteins. Samples were analyzed on an iQue Screener (IntelliCyt).

### Bio-layer interferometry (BLI)

Samples were analyzed on an Octet RED96 instrument (Pall ForteBio) using stabilization buffer (same as above) supplemented with 0.1% BSA, 20 µL/L Tween-20, and 10 μM GppNHp. Biotinylated GppNHp-loaded K-Ras WT, G12D, G12C, G12V, H-Ras WT, or N-Ras WT was immobilized onto streptavidin-coated BLI-tips (Pall ForteBio). Association was analyzed at various concentrations of SUMO-R11.1.6 or SUMO-YW1 fusion proteins (1:3 dilutions starting from 1000 nM to 1.37 nM), followed by measuring dissociation in buffer. Buffer baselines (Ras-loaded tips without addition of binder) were subtracted from the data. Dissociation constant (K_d_) values were obtained from steady-state binding analysis.

### Crystallization, diffraction data collection, and refinement

A 1:1 molar ratio of either K-Ras G12D (GppNHp) or K-Ras WT (GppNHp) and R11.1.6 was combined in stabilization buffer (same as above) and concentrated to ~20 mg/mL. Initial crystal hits were optimized from the Hampton Crystal Screen PEG/Ion suite (Hampton Research Corp). Protein crystals of the K-Ras G12D(GppNHp)/R11.1.6 complex were looped from a 2 × 2 µL hanging drop over reservoir containing 0.01 M calcium chloride, 0.01 M cadmium chloride, 0.01 M cobalt(II) chloride hexahydrate, and 15% PEG3350. Crystals of the K-Ras WT(GppNHp)/R11.1.6 complex were looped from a similar reservoir condition containing 0.03 M calcium chloride, 0.03 M cadmium chloride, 0.03 M cobalt(II)chloride, and 15% PEG3350. Crystals were cryo-protected with 10% (v/v) glycerol prior to freezing for data collection. X-Ray diffraction data were collected at Northeastern University on a Rigaku MicroMaxTM 007 HF generator and R-AXIS IV++ image plate system. Data were collected at a single wavelength (1.54 Å) and at a temperature of 100 K. Data were processed using HKL-3000R software package and structure refinement carried out in PHENIX and Coot. Both K-Ras Q61H (PDB ID 3GFT) and the solution structure of Sso7d (PDB ID 1SSO) were input as phasing models for molecular replacement. Data collection and refinement statistics for the co-crystal structures are shown in Extended Data Table 1. Ramachandran statistics for the refined K-Ras G12D/R11.1.6 and K-Ras WT/R11.1.6 structures include 97% and 99% favored backbone angles, respectively, with no outliers.

### Hydrolysis rate assay

The hydrolysis rate of both WT and K-Ras G12D was measured for the intrinsic reaction in the presence of γ^32^P-GTP, where inorganic phosphate (P_i_) release is detected using a discontinuous radiometric assay as previously published^30^. Additionally, hydrolysis rate assays were conducted for K-Ras in the presence of 400-fold excess R11.1.6, the Ras binding domains of C-Raf (residues 51–196 containing RBD and CRD), or both proteins simultaneously. The hydrolysis reaction was carried out at 37 °C and measured for a total of 360 minutes. All experiments were conducted in triplicate, and the average concentration of P_i_ was normalized to the total GTP concentration in each reaction. DynaFit4 and Prism were used for data analysis.

### Raf competition on yeast

Experiments were conducted as yeast titrations (same as above). Briefly, yeast transformed with R11.1.6 were induced in SG-CAA media overnight at 20 °C. Cells were washed in stabilization buffer (same as above) supplemented with 0.1% BSA, 20 µl/L Tween-20, and 10 μM GppNHp or GDP. K-Ras G12D or WT at 10 nM was incubated with varying concentrations of the Raf RBD (residues 51–131) from 10 μM to 3.2 nM for approximately 30 minutes. Washed cells and chicken anti-cmyc antibody were added to K-Ras/Raf and incubated for approximately 3 hours, followed by incubation with goat anti-chicken-AF 488 and anti-His-AF 647 antibodies for 20 minutes. Cells were analyzed on an iQue Screener (IntelliCyt).

### Fluorescence microscopy

HEK 293T cells were plated on #1 glass cover slips (Chemglass) and transiently transfected with EGFP-R11.1.6, EGFP-YW1, mApple-K-Ras G12D, or a combination thereof as indicated. Approximately 24 hours after transfection, cells were fixed with 4% paraformaldehyde for 10 minutes at room temperature and cover slips mounted with DAPI-containing mounting medium (Vectashield, Vector Laboratories) and dried overnight. Images were acquired at room temperature using a GE (Applied Precision) DeltaVision Spectris inverted Olympus X71 microscope with a 60x objective lens, captured with a Photometrics CoolSNAP HQ camera. SoftWoRx software was used for image acquisition, deconvolution, and co-localization analysis. EGFP signal used the ex. 475/em. 528 filter set, and mApple the ex. 632/em. 685 filter set.

### Co-immunoprecipitation assays

HEK 293T cells were transiently transfected in 10-cm plates with cmyc-R11.1.6, cmyc-YW1, HA-K-Ras G12D, or a combination thereof as indicated. Approximately 24 hours after transfection, cells were lysed in protease inhibitor (cOmplete EDTA-free protease inhibitor cocktail, Roche) containing NP-40 lysis buffer (Abcam). Whole cell lysates were analyzed by western blot for total HA-K-Ras G12D and B-Raf. Cmyc-tagged R11.1.6/YW1 were pulled down with anti-cmyc beads (Thermo Scientific) and analyzed for co-precipitation of HA-K-Ras G12D by western blotting and co-precipitation of other intracellular proteins by SDS-PAGE and silver stain (Thermo Scientific). Experiment was performed in duplicate. HA-tagged K-Ras G12D was pulled down with anti-HA beads (Thermo Scientific) and analyzed for co-precipitation of endogenous B-Raf by western blotting. Experiment was performed in triplicate.

### Cell signaling assay

HEK 293T cells were transiently transfected in 10-cm plates with cmyc-R11.1.6, cmyc-YW1, HA-K-Ras G12D, HA-K-Ras WT, or a combination thereof as indicated. Approximately 24 hours after transfection, cells were lysed in protease inhibitor (cOmplete EDTA-free protease inhibitor cocktail, Roche) containing NP-40 lysis buffer (Abcam). Whole cell lysates were analyzed by western blot for activation of MEK, ERK, and AKT with phosphospecific antibodies. Experiment was performed in triplicate. pMEK, MEK, pERK, and ERK bands were quantified using ImageJ software.

### Data availability

Atomic coordinates and structure factors for the reported crystal structures have been deposited with the Protein Data Bank (PDB): the PDB code for K-Ras WT/R11.1.6 is 5UFE and that for K-Ras G12D/R11.1.6 is 5UFQ.

## Electronic supplementary material

Supplementary information
