# Supplementary material for: An engineered protein antagonist of K-Ras/B-Raf interaction
Source: Sci Rep. 2017 Jul 19;7:5831. doi: 10.1038/s41598-017-05889-7 (PMC5517481; doi:10.1038/s41598-017-05889-7)
Supplement: Supplementary file 1 — Supplementary information [file 41598_2017_5889_MOESM1_ESM.pdf]

Supplementary Information

An engineered protein antagonist of K-Ras/B-Raf interaction

Monique J. Kauke,[1,2†] Michael W. Traxlmayr[1,2†], Jillian A. Parker[3†], Jonathan D. Kiefer[4], Ryan Knihtila[3], John McGee[5], Greg Verdine[5,6,7], Carla Mattos[3], and K. Dane Wittrup[1,2,8 *]

[1] Department of Chemical Engineering, Massachusetts Institute of Technology, Cambridge, MA 02139, USA

[2] Koch Institute for Integrative Cancer Research, Massachusetts Institute of Technology, Cambridge, MA 02139, USA

[3] Department of Chemistry and Chemical Biology, Northeastern University, Boston, Massachusetts 02115, USA

[4] Department of Chemistry and Applied Biosciences, Institute of Pharmaceutical Sciences, Swiss Federal Institute of Technology, Zurich, Switzerland

[5] Department of Chemistry and Chemical Biology, Harvard University, Cambridge, MA 02138, USA

[6] Department of Stem Cell and Regenerative Biology, Harvard University, Cambridge, MA 02138, USA

[7] Department of Molecular and Cellular Biology, Harvard University, Cambridge, MA 02138, USA

[8] Department of Biological Engineering, Massachusetts Institute of Technology, Cambridge, MA 02139, USA

† These authors contributed equally to this work.

* Corresponding author: wittrup@mit.edu

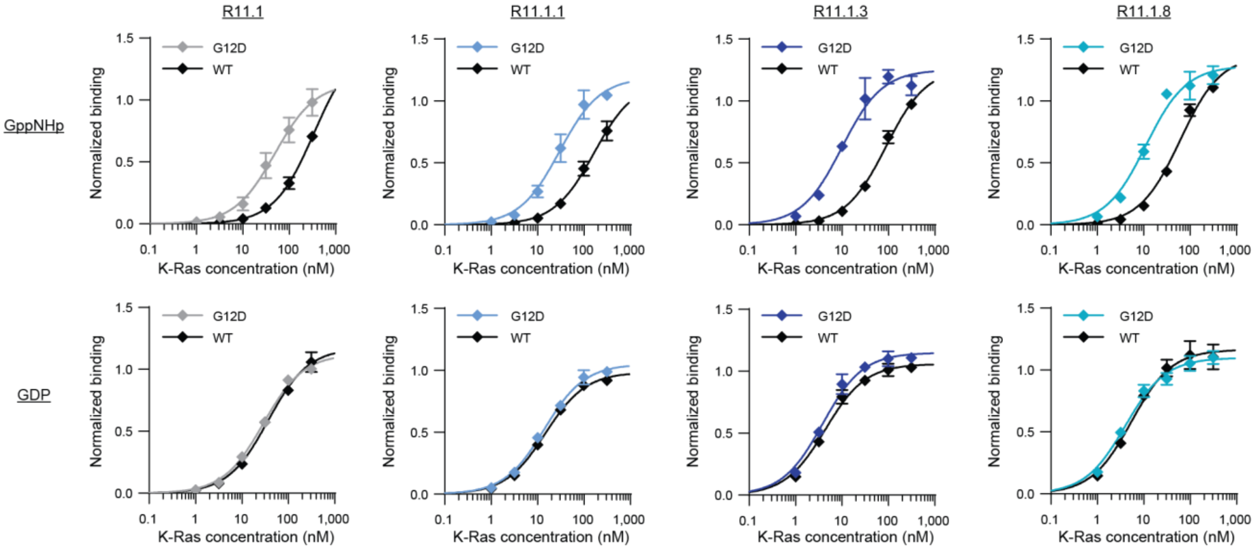

Extended Data Figure 1. Parental Sso7d R11.1 and affinity matured clones exhibit mutant-specific binding to K-Ras. Yeast surface display titrations with K-Ras loaded with GDP and the non-hydrolyzable GTP analog GppNHp. Error bars represent SEM of n = 3 independent binding experiments.

Extended Data Table 1. Data collection and refinement statistics (molecular replacement)

	K-Ras G12D(GppNHp)/R11.1.6 5UFQ	K-Ras WT(GppNHp)/R11.1.6 5UFE
**Data collection**		
Space group	C121	$P2_12_12_1$
Cell dimensions		
a, *b*, *c* (Å)	119.7, 42.7, 100.2	48.7, 64.7, 74.5
α, β, γ (°)	90, 113.3, 90	90, 90, 90
Resolution (Å)	2.20	2.30
R_{merge}	10.2	12.9
$I/\sigma(I)$	21.8 (4.5)	16.6 (5.7)
Completeness (%)	98% (94%)	98% (98%)
Redundancy	7.3 (6.5)	6.7 (7.0)
**Refinement**		
Resolution (Å)	45.17-2.20 (2.28-2.20)	40.77-2.30 (2.38-2.30)
No. reflections	173,684 (14,914)	71,673 (7,517)
R_{work} / R_{free}	20.0/26.3	18.4/24.1
No. atoms	3702	1882
Protein	3510	1733
Ligand/ion	74	46
(specify/describe)		
Water	118	95
B factors	41.9	22.3
Protein	42.1	22.4
Ligand/ion	31.2	18.3
Water	40.9	22.4
R.m.s. deviations		
Bond lengths (Å)	0.009	0.008
Bond angles (°)	1.15	1.06

[a] Values in parentheses are for highest-resolution shell.

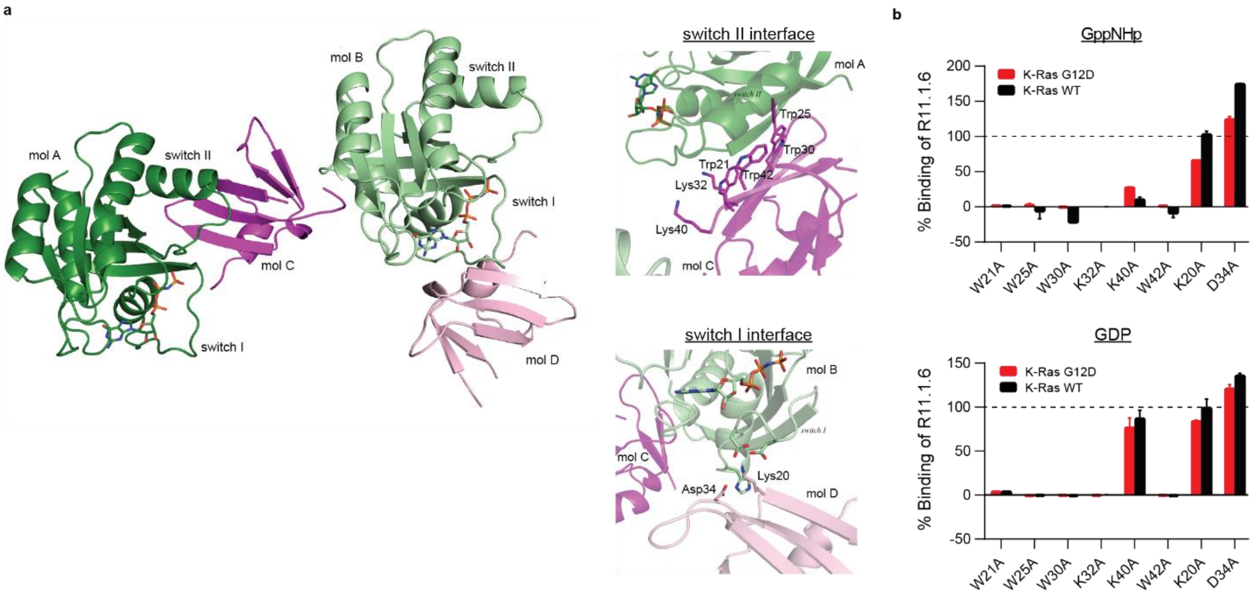

Extended Data Figure 2. R11.1.6 binds switch II of K-Ras G12D. a, Global view of the R11.1.6/K-Ras G12D co-crystal structure, containing four molecules in the asymmetric unit. Molecules A (K-Ras G12D, green) and C (R11.1.6, magenta) interact at the switch II region, while molecules B (K-Ras G12D, green) and D (R11.1.6, magenta) bind at switch I. On the right, major residues involved in each binding interaction are indicated. **b**, Binding as measured by yeast surface display of R11.1.6 mutants of the residues involved in both switch I and switch II interactions indicated in **a** to K-Ras (10 nM). Binding has been normalized to non-mutated R11.1.6 binding. Error bars represent SEM of n = 3 binding experiments.

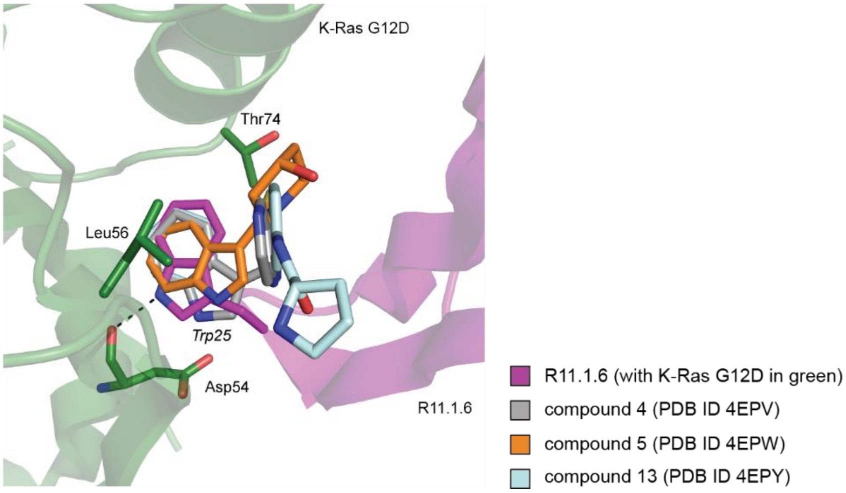

Extended Data Figure 3. R11.1.6 exhibits similar binding to small molecules against K-Ras. Overlay of the co-crystal structure of R11.1.6 with K-Ras G12D with structures of K-Ras in complex with small molecules compound 4 (PDB ID 4EPV), compound 5 (PDB ID 4EPW), and compound 13 (PDB ID 4EPY)[14], showing the striking similarity in binding position of the indole moieties of the small molecules and the R11.1.6 Trp25 residue. K-Ras G12D residues are indicated in regular font.

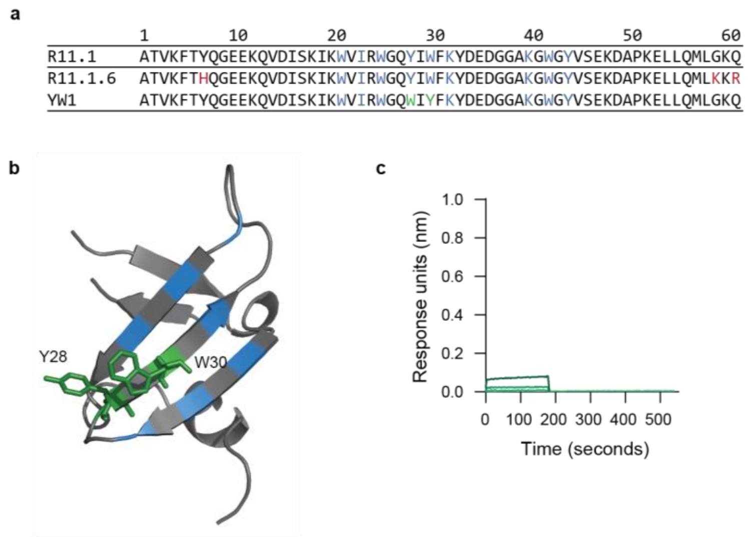

Extended Data Figure 4. Scrambling of key R11.1.6 residues prevents binding to K-Ras. a, Amino acid sequences of parental binder R11.1, affinity-matured R11.1.6, and scrambled control YW1. The nine residues of the Sso7d binding surface are depicted in blue; R11.1 framework mutations are shown in red. The two swapped residues of YW1 are indicated in green. **b**, Crystal structure of R11.1.6, in which residues are colored as in **a**, showing Y28 and W30 which are swapped in YW1. The C-terminus is slightly disordered. **c**, Binding of YW1 to immobilized GppNHp-loaded K-Ras G12D measured using bio-layer interferometry. Concentrations of YW1 curves from dark to light: 1000, 333.3, 111.1, 37, 12.3, 4.1, 1.4 nM.

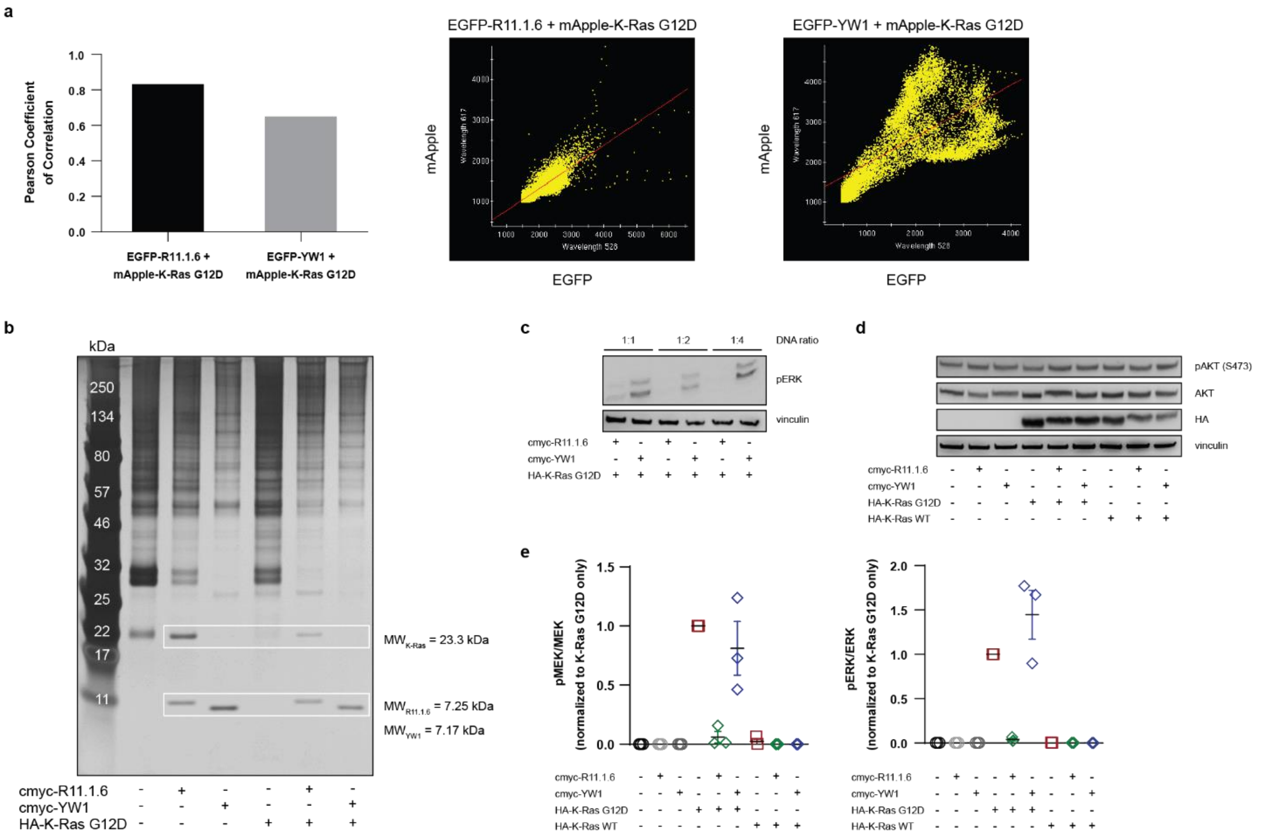

Extended Data Figure 5. R11.1.6 co-localizes with K-Ras G12D in cells, specifically pulls down K-Ras, shows concentration-dependent inhibition of MAPK signaling, but does not affect the PI3K cascade. a, Pearson coefficients of correlation and the respective scatter plots obtained from co-localization quantification of the merged images shown in Figure 4a. **b**, Silver stained gel of co-immunoprecipitation of cmyc-R11.1.6 in co-transfected HEK 293T cells. Anti-cmyc beads pulled down cmyc-R11.1.6 and cmyc-YW1 at the proper molecular weight (MW). Only cells transfected with R11.1.6 co-immunoprecipitate K-Ras. Higher molecular weight bands show non-specificity of anti-cmyc beads, apparent from the mock transfected lane. Results are representative of n = 2 biological replicates. **c**, Concentration dependence of R11.1.6-mediated inhibition of phosphorylated ERK (pERK) in co-transfected HEK 293T cells. Increasing R11.1.6 transfected DNA reduces pERK (DNA ratio, K-Ras G12D:R11.1.6). Results are representative of

n = 3 biological replicates. **d**, Effect of R11.1.6 on phosphorylation of endogenous AKT (pAKT) at serine 473 via HA-K-Ras G12D-induced signaling in co-transfected HEK 293T cells, showing lack of inhibition of signaling by R11.1.6. Results are representative of n = 3 biological replicates. Full-length blots of the cropped ones shown here are given in Extended Data Figures 8 and 9. **e**, Quantification of pMEK/MEK and pERK/ERK signals of the three biological replicate blots, as represented by the images shown in Figure 4d. Data were normalized to the K-Ras G12D only transfected samples.

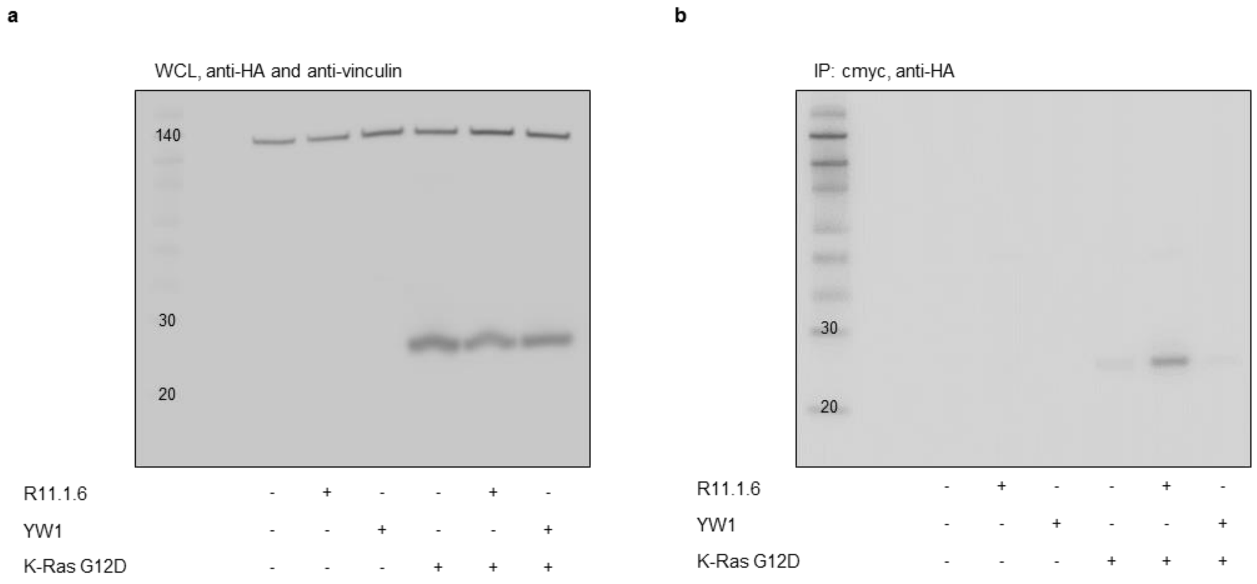

Extended Data Figure 6. Full western blots of Figure 4b. a, Western blot of whole cell lysate (WCL) of HA-tagged K-Ras G12D with cmyc-R11.1.6/YW1 co-transfected HEK 293T cells. **b**, Western blot of cmyc immunoprecipitated samples of HEK293T cells co-transfected with HA-tagged K-Ras G12D and cmyc-R11.1.6/YW1. Results are representative of n = 2 biological replicates. Molecular weights in kDa are indicated.

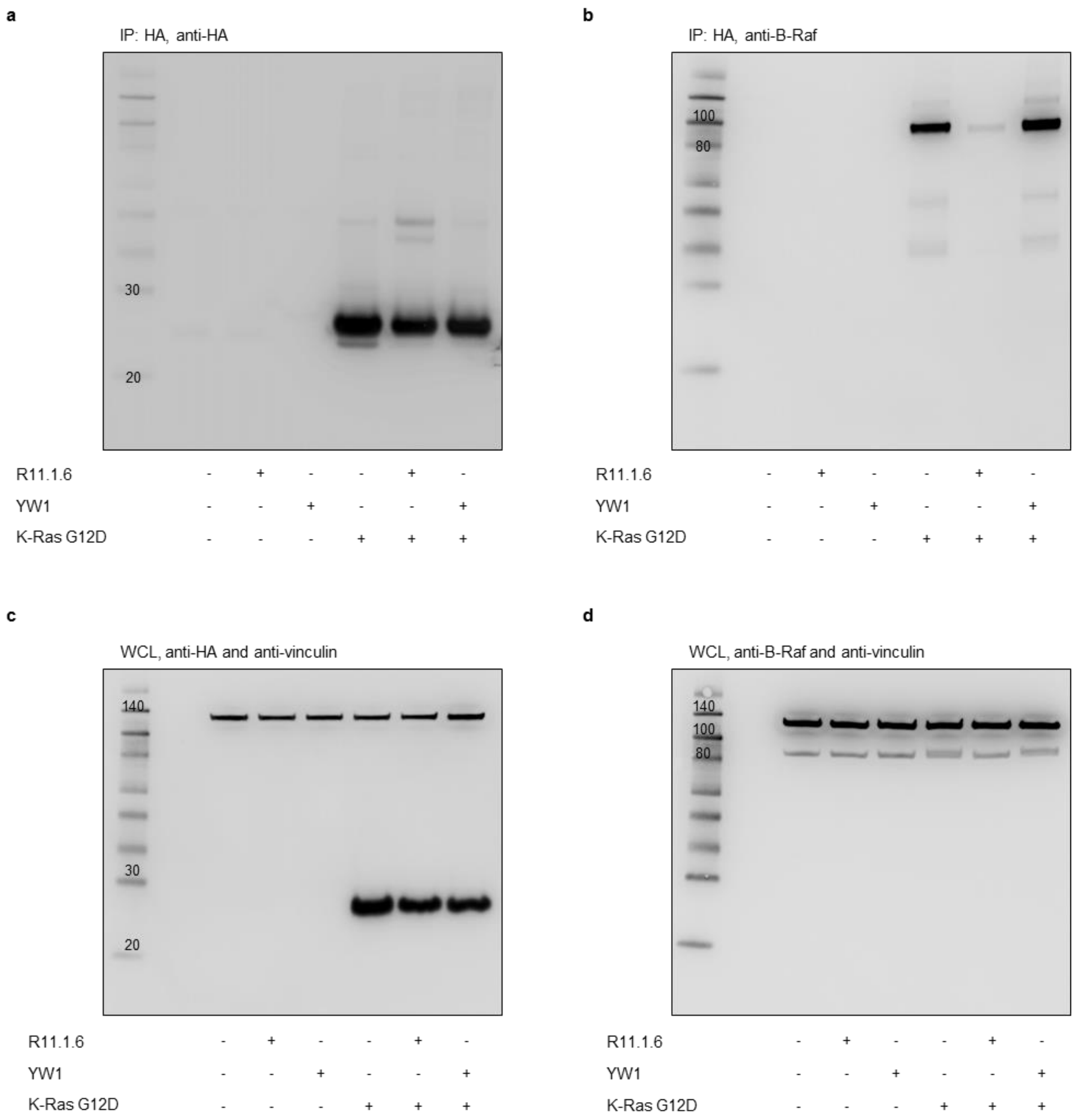

Extended Data Figure 7. Full western blots of Figure 4c. a-b, Western blots of HA immunoprecipitated samples of HEK293T cells co-transfected with HA-tagged K-Ras G12D and cmyc-R11.1.6/YW1. **c-d**, Western blots of whole cell lysate (WCL) of HA-tagged K-Ras G12D with cmyc-R11.1.6/YW1 co-transfected HEK 293T cells. Results are representative of n = 3 biological replicates. Molecular weights in kDa are indicated.

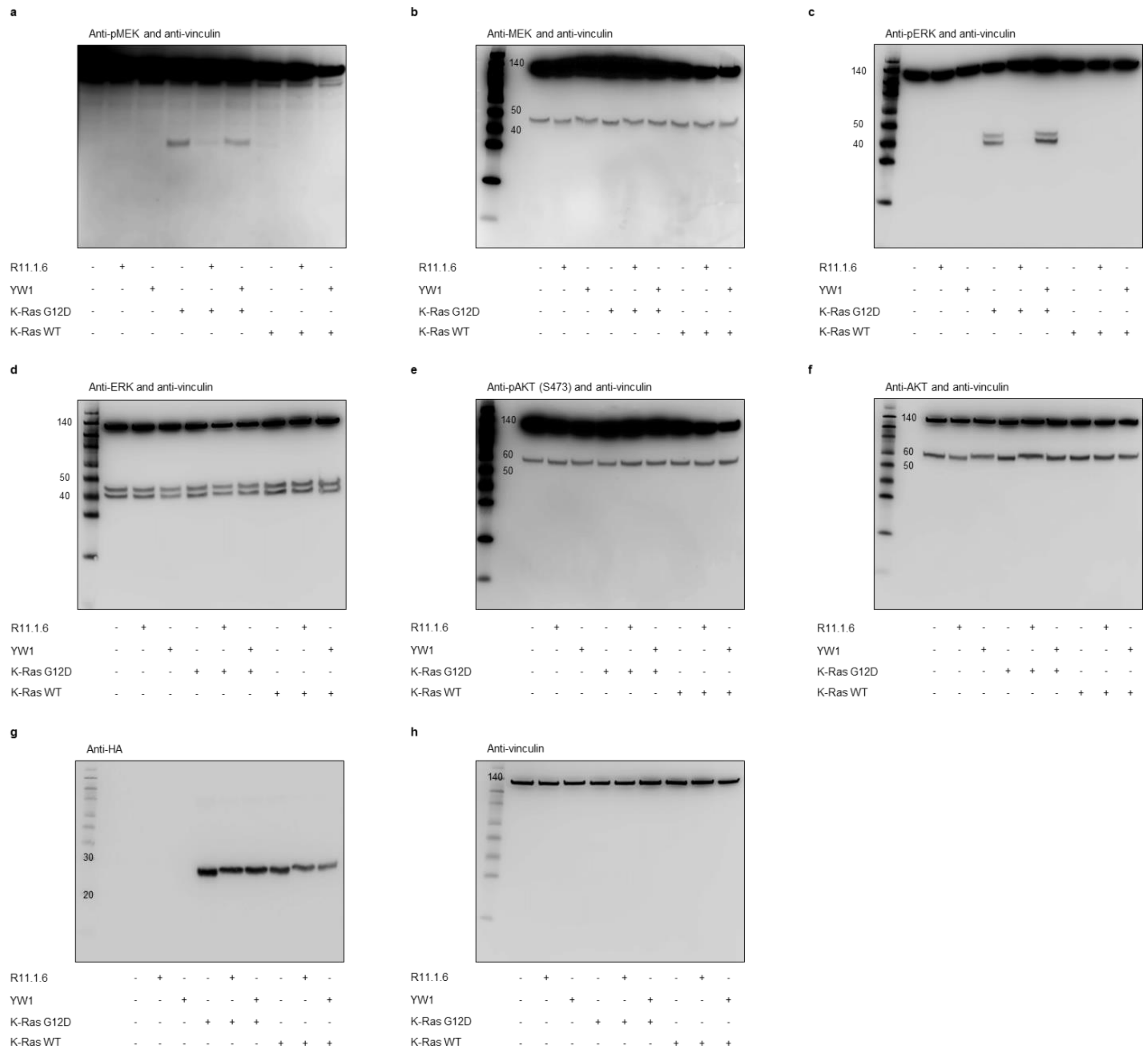

Extended Data Figure 8. Full western blots of Figure 4d and Extended Data Figure 5d. a-h, Western blots of whole cell lysates of HEK293T cells co-transfected with HA-tagged K-Ras G12D/WT and cmyc-tagged R11.1.6/YW1. Results are representative of n = 3 biological replicates. Molecular weights in kDa are indicated.

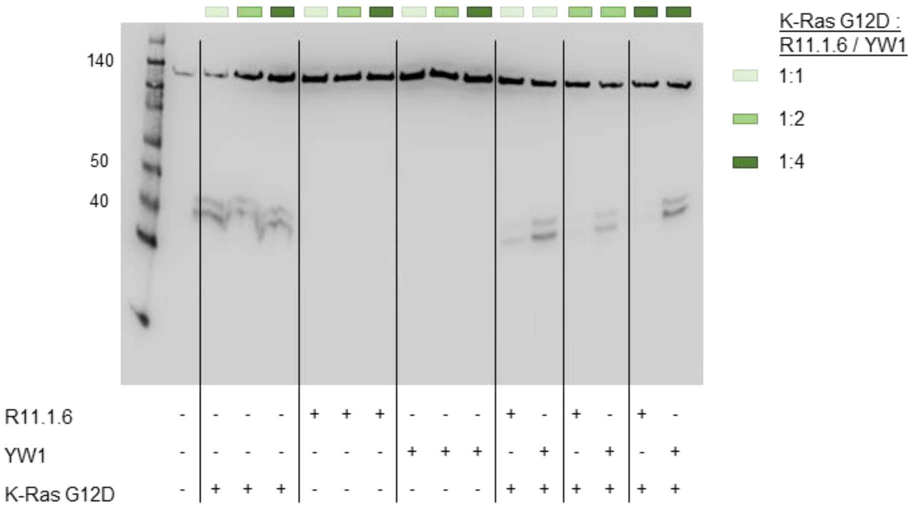

Extended Data Figure 9. Full western blot of Extended Data Figure 5c. Western blot against vinculin and pERK of whole cell lysates of HEK293T cells co-transfected with varying DNA ratios (indicated by green bars) of HA-tagged K-Ras G12D and cmyc-tagged R11.1.6/YW1. Results are representative of n = 3 biological replicates. Molecular weights in kDa are indicated.
